# Motivational readiness for physical activity and health literacy: results of a cross-sectional survey of the adult population in Germany

**DOI:** 10.1186/s12889-023-15219-4

**Published:** 2023-02-14

**Authors:** Maike Buchmann, Susanne Jordan, Anne-Kathrin Mareike Loer, Jonas David Finger, Olga Maria Domanska

**Affiliations:** 1 Department of Epidemiology and Health Monitoring, Robert Koch Institute, Nordufer 20, 13353 Berlin, Germany; 2 Senate Department for Higher Education and Research, Long-Term Care and Gender Equality, Department of Health, Oranienstraße 106, 10969 Berlin, Germany

**Keywords:** Health literacy, Motivational readiness, Stages of change, Transtheoretical model, Physical activity, Health behaviour change, Prevention, Health promotion, Cross-sectional study, German Health Update

## Abstract

**Background:**

Health literacy, defined as the knowledge, motivation, and competences to use health information to improve health and well-being, is associated with regular physical activity. However, there is limited evidence on whether health literacy is also related to the motivational readiness for physical activity in a general population. The aim of this study was to investigate whether motivational readiness for leisure-time physical activity is associated with health literacy.

**Methods:**

Analyses were based on data of 21,895 adults from the cross-sectional German Health Update and European Health Interview Survey 2014/2015 (GEDA 2014/2015-EHIS). Motivational readiness for leisure-time physical activity was assessed with stages of change for physical activity with a set of validated items. It was then classified, according to an established algorithm, into five stages: precontemplation, contemplation, preparation, action, and maintenance. Health literacy was measured with the short form of the European Health Literacy Survey Questionnaire (HLS-EU-Q16) and categorised as low, medium, and high. For bivariate and multinomial logistic regression analyses, the stages were categorised in three phases as: (1) *no intention* (precontemplation), (2) *planning* (contemplation or preparation), and (3) *in activity* (action or maintenance). The models were adjusted for sex, age, education, health consciousness, self-efficacy, and self-perceived general health status.

**Results:**

High compared to low health literacy was associated with a 1.65-times (95% CI = 1.39–1.96) greater probability of being *in activity* than *planning*. High compared to low health literacy was associated with a reduced risk of having no intention to change physical activity behaviour (relative risk ratio, RRR = 0.84, 95% CI = 0.75–0.95). The associations persisted after adjusting for covariates.

**Conclusion:**

High health literacy was positively associated with more advanced phases of motivational readiness for leisure-time physical activity. Therefore, taking health literacy into account in interventions to promote motivational readiness for leisure-time physical activity could be a useful approach.

## Background

Physical activity is considered to yield substantial health benefits including reducing the risk of overall mortality [1, 2]. Evidence shows an inverse relationship between physical activity and metabolic syndrome as well as cardiovascular disease [3, 4]. Furthermore, physical activity is associated with reduced incidence and better outcomes in cancer [5, 6]. Being physically active is also linked to a lower risk for depression [7].

However, a large proportion of the population in Germany and worldwide does not achieve the health promoting levels of physical activity recommended by the World Health Organization (WHO), consisting of at least 150 min of moderate aerobic activity or 75 min of vigorous aerobic activity or an adequate combination of both as well as two sessions of muscle strengthening exercises per week [8]. In terms of aerobic activity, a pooled analysis of more than 300 surveys from 168 countries found an age-standardised prevalence of physical inactivity of 27.5% globally and 42.3% in high-income Western countries [9]. The results for Germany are in line with this: depending on the definition of the indicator, more than half of women (57.4%) and men (51.2%) do not reach the aerobic physical activity recommendations and about four out of five women (79.5%) and three out of four men (75.3%) do not reach both the recommendations for aerobic physical activity and muscle strengthening [10]. Motivating people who are not sufficiently active or not active to become more active is a major challenge in public health promotion. In particular, encouraging physical activity during leisure time represents a promising approach, as evidence suggests that the health-promoting effect of physical activity during leisure time exceeds that of work-related and transportation-related activities [11–13]. Developing the habit of regular physical activity cannot be summed up as an ‘all-or-nothing phenomenon’, but is a complex process with a temporal dimension of stages of behavioural change, which is considered in the concept of motivational readiness for physical activity [14, 15]. It can be assumed that the part of the population that is less active in leisure-time, is heterogenous and differs in their motivational readiness for physical activity [16, 17]. One approach to enhance motivational readiness for leisure-time physical activity, could be to promote health literacy, as it has been shown to be associated with physical activity.

Health literacy is a concept that has gained increasing importance in international public health research and policy since the 1990s [18, 19]. While the concept initially focused on functional health literacy in terms of the ability to read and understand health information, including medical terms, a broader definition usually underlies today’s research and interventions [20, 21]. This comprises the knowledge, competences, and motivation to access, understand, assess, and apply health information to maintain and improve health and well-being regarding health care, disease prevention, and health promotion [20, 22]. While studies assume that between 44.2% and 58.8% of the adult population in Germany have low health literacy levels [23–25], health literacy is considered as a modifiable factor [19]. As research indicates that higher health literacy is associated with better health status and favourable health behaviour [23, 24, 26–28], it is a promising target for health promoting interventions. The promotion of health literacy aims at empowering individuals to promote their own and others’ health. Furthermore, it is conceptualized in research and practice that health literacy goes beyond personal skills, such as knowledge about health risks. It also reflects the complex societal demands for a healthy life, for example a lack of understandable information on health risks and limited access to opportunities to be physically active [19]. It is known from a number of empirical studies that health literacy is positively associated with physical activity [26, 29–31], but it is not clear whether it also is associated with motivational readiness for physical activity. If this is the case, health literacy promotion could be used to enhance motivational readiness for physical activity.

To understand motivational readiness, the concept of stages of change for physical activity is often used, which is the core construct of the transtheoretical model of behavioural change. This model was originally developed by Prochaska and DiClemente in the context of smoking cessation in 1983 [32]. It was then applied to physical activity by Marcus et al. [14] and validated in multiple studies [33, 34]. According to the model, the course of behavioural change can be conceptualized as a transition through the stages of *precontemplation*, *contemplation*, *preparation*, *action*, and *maintenance* [32]. These stages are linked to different patterns of experiential and behavioural processes of change, for example, the process of consciousness raising (gathering information about the behaviour), which facilitates the transition from the initial to the later phases [35]. In the context of physical activity, the stages of change are used to categorise individuals’ readiness for behaviour change into categories ranging from having no intention to change physical activity to maintaining physical activity [14, 33]. The assumption of the transtheoretical model is that interventions aimed at changing behaviour can be tailored to different stages of motivational readiness [36] and thus have a higher effectiveness than ‘one-size-fits-all’ solutions. Evidence on the effectiveness of stage-matched physical activity promoting interventions in adult populations has shown inconsistent results so far: a systematic review on the effectiveness of interventions to improve physical activity using the transtheoretical model included 11 studies, but only five showed a positive effect; these were characterized by participants being at early stages and by personal consultations [37]. Further detailed research on the association between motivational readiness for physical activity and health literacy is needed for informed planning of such interventions [38].

The assumption of an association between health literacy and motivational readiness to change physical activity behaviour is based on theoretical models [20, 39, 40] and the first empirical findings [41]. The theoretical framework by von Wagner et al. on health literacy and health actions assumes that health literacy influences motivational and volitional processes [39]. As health literacy comprises not only knowledge (cognitive dimension) and competence (behavioural dimension) but also the motivation (conative-affective dimension) to apply information in a health-promoting way [42], it is conceptually linked to motivational readiness for physical activity. According to the transtheoretical model of behaviour change, cognitive and behavioural processes, such as consciousness raising and decisional balance (balancing benefits and costs) [32, 43, 44], determine the progression through the stages of change. These processes could be described as processes of actively dealing with information on health behaviour which matches the concept of health literacy. Despite the conceptual link, there is only limited empirical evidence for an association between health literacy and motivational readiness for physical activity so far. To the best of our knowledge, the relationship between health literacy and the stages of change for physical activity has only been explored explicitly in a study with a regional adult sample in Turkey (n = 826) [41]. Aygun and Cerim found that participants with higher health literacy scores were found to be in more advanced stages of change with respect to general health behaviours, including exercising [41]. A study in young men [45] showed that low health literacy is associated with avoiding thoughts about exercise, whereas information seeking is linked to more advanced stages of change for physical activity. Some studies regarding other health behaviours point in the same direction, indicating a positive association between health literacy and the stages of change, for example, for smoking cessation [46] and glycaemic control in diabetes [47]. Overall, there is theoretical and empirical support for assuming that health literacy and motivational readiness for physical activity are associated. The aim of this study was to investigate whether different phases of motivational readiness for leisure-time physical activity are associated with health literacy.

## Methods

### Study design and participants

This study used data of the cross-sectional German Health Update and European Health Interview Survey (GEDA 2014/2015-EHIS) [48]. To obtain representative data of the German-speaking adult population, data was collected using a two-stage cluster sampling approach. Firstly, 301 sample points across Germany were selected randomly based on the sizes of the respective federal state and municipalities. Secondly, a random sample was drawn from the local population registers of each sample point. Between November 2014 and July 2015, 92,771 adults were invited to respond by filling out an online questionnaire. If they did not answer within four weeks, they were reminded via mail and offered to either participate online or with a paper-based questionnaire. The method is described in detail elsewhere [48]. The survey was approved by the Federal Commissioner for Data Protection and Freedom of Information of Germany [48]. All respondents gave informed written consent before enrolling for the survey after being thoroughly informed about the survey’s objectives and data protection. A total of 24,016 participants, aged 18 years and older, took part in the survey, resulting in a response rate of 26.9% [48].

### Outcome variable

#### Stages of change for physical activity

The motivational readiness for physical activity was assessed with the internationally used and validated instrument *stages of change for physical activity* [44, 49, 50], which was adapted for the questionnaire of the GEDA 2014/2015-EHIS [48]. The assessment was conducted through two successive sets of questions which form the basis for an algorithm to map the stages of behaviour change for physical activity.

First, leisure-time physical activity was assessed with three questions of the validated German version of the leisure-time physical activity domain of the European Health Interview Survey – Physical Activity Questionnaire (EHIS-PAQ) [10]. It differentiates between ‘aerobic’ and ‘muscle-strengthening’ activities. The participants were asked on how many days and for how long in total they engage in physical activities of at least moderate intensity in their leisure time for at least ten minutes at a time in a typical week. In addition, they reported on how many days in a typical week they did muscle strengthening exercises [10, 51]. Based on the answers, participants were categorised in two groups: ‘not active’ or ‘active’ during leisure time. The following condition had to be fulfilled in order to be classified in the ‘active during leisure time’ group: engaging in at least 150 min of at least moderate-intensity aerobic physical activity per week and doing recreational muscle-strengthening exercises on at least two days per week during leisure time. These criteria are oriented towards the WHO recommendations for physical activity [1, 8]. We focused on leisure-time activity because its beneficial effects on health exceed those of work-related and transportation-related physical activity [11–13]. Furthermore, in the design of the questionnaire the questions on the motivational readiness refer specifically to leisure-time physical activity.

Second, three items on the motivational readiness for physical activity were asked. The aim was to determine the existence of an intention to change, the intentional start of a change, and if applicable, the duration of the current leisure-time physical activity:

1. ‘We have already asked you about the frequency and duration of physical and sporting activity in your leisure time in a typical week. For how many months have you been physically active or inactive in this way?’ (Answer options: ‘less than 6 months’ or ‘6 months or more’).
2. ‘Do you plan to be physically active more often than before?’ (Answer options: ‘yes’ or ‘no’).
3. In case of a positive answer to the second question, the following question was asked: ‘When do you plan to be physically active more often than before?’ (Answer options: ‘in the next few months’ [in which case the number of months should be indicated] or ‘in the next 30 days’).

**Fig. 1:**
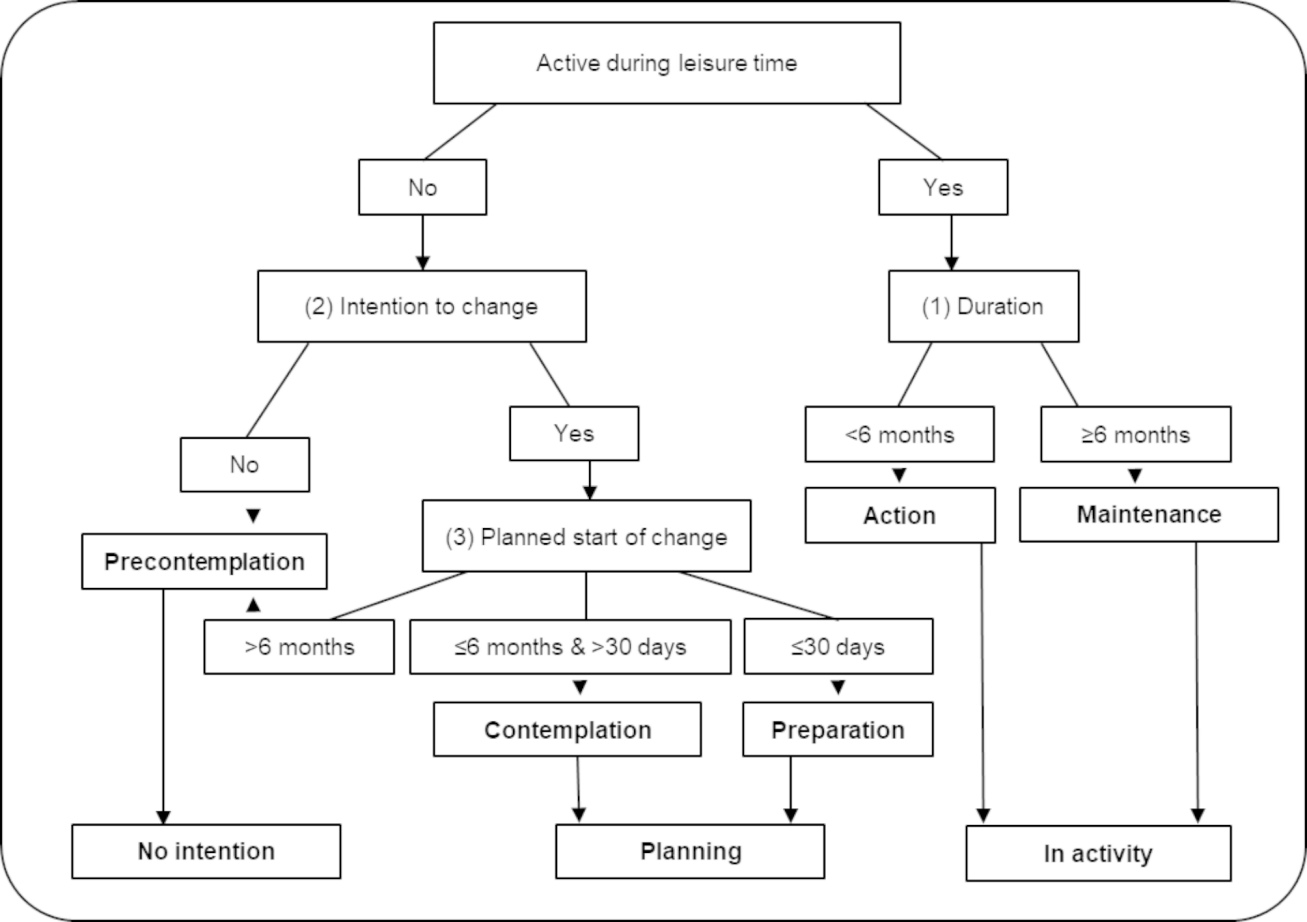
Algorithm to determine stages of change for physical activity, adapted from Ronda et al. [52]

The assignment of participants to a stage of change for physical activity (i.e., to the stages of precontemplation, contemplation, preparation, action, or maintenance) was achieved by using a slightly adapted established algorithm [44, 52]. As we were primarily interested in the effect of health literacy on the probability of being in earlier or more advanced stages of change for physical activity, these five stages were combined into three phases: *no intention* (precontemplation), *planning* (contemplation and preparation), and *in action* (action and maintenance) (see Fig. 1).

### Predictor variable

#### Health literacy

Health literacy was assessed using the validated short form of the European Health Literacy Survey Questionnaire (HLS-EU-Q16) with 16 items [53, 54] used in many studies in different countries and contexts [55–58]. The instrument is based on a multidimensional understanding of health literacy and covers the dimensions of finding, understanding, assessing, and applying information in the domains of disease management, prevention, and health promotion [20]. The HLS-EU-Q16 measures self-reported difficulties in dealing with health information in the context of existing social demands and structures. Using a four-point Likert scale ranging from ‘very easy’ to ‘very difficult’, participants responded to questions about health information in everyday situations and health care. Of 16 items only two refer also to physical activity: ‘On a scale from very easy to very difficult, how easy or difficult do you find it to understand health warnings about behaviours such as smoking, low physical activity, or excessive drinking?’ (item no. 9) and ‘How easy would you say it is to assess which everyday habits are related to your health (drinking and eating habits, physical activity, etc.)?’ (item no. 16). The other questions were about, for example, finding information about treatments, understanding why screening is important, or following the doctor’s or pharmacist’s instructions. In line with Röthlin et al. [54], the answers were dichotomised (‘very easy’ and ‘fairly easy’ = 1; ‘fairly difficult’ and ‘very difficult’ = 0). If at least 14 out of 16 items were answered, a sum score was calculated. Following Röthlin et al. [54], categories were generated, namely low (sum score: 0–8), medium (sum score: 9–12), and high (sum score: 13–16) health literacy; the category wording slightly differs, by being more descriptive than evaluative (e.g., low instead of inadequate health literacy).

### Covariates

Several socio-demographic variables were included as covariates, as empirical evidence suggests a link with motivational readiness for physical activity [49, 59]. Sex (women/men) and age (collected in full years and categorized in four age groups [in years: 18–29, 30–44, 45–64, and 65+]) were considered. The level of education was collected with questions about the highest educational degree and professional qualification. According to the International Standard Classification of Education 1997, three educational levels (low, medium, and high) were distinguished [60].

As psychosocial factors, health consciousness and self-efficacy were considered. Health consciousness is considered to be associated with participation in prevention measures [61]. It was assessed with one question from the health consciousness scale according to Gould [62]: ‘How much care do you usually take of your health?’ [62]. Participants could respond on a five-level Likert scale ranging from ‘very much’, ‘much’, ‘to an average degree’, ‘not so much’, to ‘not at all’. For analysis, the variable was dichotomised into ‘to an average degree/not so much/not at all’ and ‘much/very much’. Self-efficacy is conceptualized as an important predictor of behavioural change within the transtheoretical model and evidence supports it being associated with motivational readiness to change physical activity behaviour [14, 36]. Self-efficacy was self-reported using the validated general self-efficacy short scale (Allgemeine Selbstwirksamkeit Kurzskala, ASKU) [63] through three statements about the general ability to solve tasks [63], for example, ‘In difficult situations I can rely on my own abilities’ (answer options: strongly agree, agree, neutral, disagree, strongly disagree). To evaluate the individual extent of self-efficacy, the mean value of the answers (between 5 = fully agree and 1 = strongly disagree) of the three items was calculated in line with the evaluation scheme suggested by the authors of the ASKU [63].

Data about the self-perceived general health status was considered due to the known correlation between health status and being physically active [64, 65]. It was collected with the first question on self-perceived health within the Minimum European Health Module [66]: ‘How is your health in general?’. Answer options were ‘very good’, ‘good’, ‘fair’, ‘poor’, and ‘very poor’ and classified into two categories as ‘good/very good’ and ‘fair/poor/very poor’.

### Statistical analysis

The analyses were conducted with a weighting factor that corrects for deviations between the sample and the structure of the German population (as of 31 December 2014) in terms of gender, age, education, and community type (degree of urbanisation). After descriptive analyses of the outcome variable stages of change for physical activity and the predictor variable health literacy, bivariate analyses were performed between the outcome stages of change for physical activity and health literacy and the covariates. Chi-square tests were used to identify associations. Then, crude and multivariable adjusted analyses were conducted through multinomial logistic regression. This statistical approach can be used in polytomous outcomes (dependent variables with more than two levels) [49, 59] as is the case with the three phases in the underlying operationalisation of motivational readiness for physical activity. The *planning* phase was defined as the reference category to identify the probability (relative risk ratio, RRR) to be in the less (*no intention*) or more advanced phase (*in activity*) rather than in the planning phase, according to the level of health literacy. Four models were used: Model 1 investigated the crude association between health literacy and the motivational readiness for physical activity. Model 2 corresponds to Model 1, except that it was adjusted for socio-demographic variables (age, sex, and education). Model 3 was adjusted additionally for psychosocial variables (health consciousness and self-efficacy). Model 4 was further adjusted for self-perceived general health status. All analyses were performed using Stata (version 15.1) [67].

## Results

Participants with valid data for all variables considered were eligible for the analysis (complete case analysis, n = 21,895). Socio-demographic characteristics and health literacy of the study population are presented in Table 1 and their weighted frequencies correspond to an expected distribution of the population in Germany. Almost two thirds of the population showed a high level of health literacy (63.5%, 95% CI = 62.6–64.3), 26.4% a medium level (95% CI = 25.7–27.2), and 10.1% a low level (95% CI = 9.6–10.7).

**Table 1:** Socio-demographic characteristics and health literacy of study participants, n = 21,895

	n^1^	%^2^
**Sex**		
Women	11,894	50.1
Men	10,001	49.9
**Age groups** (in years)		
18–29	3,718	17.8
30–44	5,029	23.0
45–64	8,369	37.5
65+	4,779	21.8
**Education**		
Low	3,011	17.6
Medium	11,310	59.1
High	7,574	23.3
**Health Literacy**		
Low	2,018	10.1
Medium	5,673	26.4
High	14,204	63.5

^1^ = unweighted, ^2^ = weighted

**Fig. 2:**
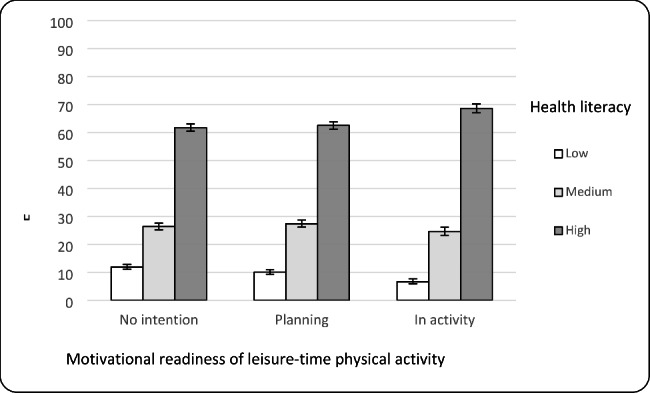
Motivational readiness for physical activity by health literacy, n=21,895

Only about one fifth of the population (21.3%, 95% CI = 20.6–22.1) was classified as active in terms of the chosen criterion to define leisure-time physical activity (150 min of at least moderate physical activity during leisure time and at least two days of muscle-strengthening activity per week). This proportion corresponds to the proportion of the population in the *in activity* phase. The majority were in the phases *no intention* (40.4%, 95% CI = 39.4–41.4) or *planning* to change physical activity (38.3%, 95% CI = 37.5–39.0) (Table 2). Bivariate analyses between the phases of motivational readiness for physical activity and health literacy showed that the proportion with high health literacy was the highest in the phase *in activity* compared to the other phases. Conversely, the group *no intention* showed the highest proportion with low health literacy (Fig. 2).

**Table 2:** Characteristics of motivational readiness for physical activity, n = 21,895 (bivariate analyses)

Phases of motivational readiness for physical activity	No intention		Planning		In activity		p-value
	%	(95% CI)	%	(95% CI)	%	(95% CI)	
**Total**	40.4	(39.4–41.4)	38.3	(37.5–39.0)	21.3	(20.6–22.1)	
**Health literacy**							< 0.001
Low	11.9	(11.1–12.8)	10.1	(9.3–11.0)	6.7	(5.9–7.7)	
Medium	26.4	(25.2–27.6)	27.4	(26.2–28.7)	24.6	(23.2–26.1)	
High	61.7	(60.4–63.1)	62.5	(61.1–63.8)	68.6	(67.0–70.2)	
**Sex**							< 0.001
Women	50.7	(49.4–52.0)	51.7	(50.4–52.9)	46.0	(44.5–47.5)	
Men	49.3	(48.0–50.6)	48.3	(47.1–49.6)	54.0	(52.5–55.5)	
**Age groups** (in years)							< 0.001
18–29	10.8	(9.9–11.7)	21.1	(20.0–22.1)	25.0	(23.6–26.5)	
30–44	19.5	(18.5–20.4)	28.8	(27.7–30.0)	19.0	(17.9–20.3)	
45–64	38.5	(37.5–39.5)	37.1	(36.0–38.3)	36.2	(34.6–37.9)	
65+	31.2	(30.1–32.4)	13.0	(12.2–13.9)	19.7	(18.4–21.1)	
**Education**							< 0.001
Low	20.8	(19.5–22.2)	15.7	(14.6–16.9)	15.0	(13.7–16.5)	
Medium	58.9	(57.4–60.4)	59.6	(58.1–61.1)	58.5	(56.8–60.2)	
High	20.2	(19.2–21.4)	24.7	(23.4–26.1)	26.5	(24.9–28.1)	
**Health consciousness**							< 0.001
To an average degree/not so much/not at all	55.2	(53.8–56.6)	60.3	(59.0–61.6)	33.5	(32.0–35.1)	
Much/very much	44.8	(43.4–46.2)	39.7	(38.4–41.0)	66.5	(64.9–68.0)	
**Self-efficacy** ^**1**^							< 0.001
Mean	4.0	(4.0–4.1)	4.1	(4.1–4.1)	4.2	(4.2–4.2)	
**Health status**							< 0.001
Fair/poor/very poor	34.6	(33.3–35.8)	31.3	(30.2–32.5)	20.2	(18.8–21.7)	
Good/very good	65.4	(64.2–66.7)	68.7	(67.5–69.8)	79.8	(78.3–81.2)	

^1^ Self-efficacy is indicated as a continuous variable using the mean score of the general self-efficacy short scale and the standard deviation (SD).

All of the covariates were distributed differently with regard to the outcome variable motivational readiness for leisure-time physical activity (Table 2). In the *in activity* phase, higher proportions of men, 18–29-year-olds, those with a high health consciousness, and a ‘good’ or ‘very good’ health status were shown compared to the other phases of motivational readiness. In contrast, the over-65-year-olds, those with low education, and with a poorer health status were more frequently represented in the *no intention* phase than in the other phases. In the *planning* phase, the highest proportions of 30–44-year-olds and those with lower health consciousness (care ‘to an average degree’ or less for health) were found compared to the other phases. The self-efficacy score was higher in higher stages of motivational readiness.

The crude and adjusted results of the multinomial regression models for motivational readiness for physical activity and health literacy are presented in the following paragraphs.

### No intention (reference group: planning)

According to crude analysis (Model 1; shown in Table 3a), participants with medium (RRR 0.82) and high (RRR 0.84) health literacy were less likely to be in the *no intention* group rather than the *planning* group. This means that the probability of being in the *no intention* group was lower with a higher level of health literacy. This association was also significant after controlling for sex, age, education, health consciousness, self-efficacy, and health status, and no major changes in the RRR occurred (Model 4; shown in Table 3a). Those participants with high or medium health literacy compared to those with low health literacy were 17% respectively 15% less likely to be in the *no intention* group compared to the *planning* group.

**Table 3a:** Motivational readiness for leisure-time physical activity according to health literacy and the covariates for “No intention” ^1^, n = 21,895

	Model 1	Model 2	Model 3	Model 4
	RRR (95% CI)	RRR (95% CI)	RRR (95% CI)	RRR (95% CI)
**Health literacy**				
Low	Ref.	Ref.	Ref.	Ref.
Medium	0.82** (0.72–0.93)	0.86* (0.76–0.98)	0.87* (0.76–0.99)	0.85* (0.75–0.98)
High	0.84** (0.75–0.95)	0.84** (0.75–0.95)	0.86* (0.76–0.97)	0.83** (0.73–0.93)
**Sex**				
Women		Ref.	Ref.	Ref.
Men		1.12** (1.04–1.22)	1.14** (1.05–1.23)	1.14** (1.05–1.23)
**Age group** (in years)				
18–29		0.71*** (0.62–0.81)	0.71*** (0.62–0.81)	0.70*** (0.61–0.79)
30–44		Ref.	Ref.	Ref.
45–64		1.53*** (1.39–1.69)	1.52*** (1.38–1.68)	1.59*** (1.44–1.75)
65+		3.43*** (3.02–3.89)	3.35*** (2.95–3.81)	3.65*** (3.20–4.16)
**Education**				
Low		Ref.	Ref.	Ref.
Medium		0.79*** (0.71–0.88)	0.79*** (0.71–0.89)	0.78*** (0.70–0.88)
High		0.63*** (0.57–0.71)	0.63*** (0.56–0.71)	0.61*** (0.54–0.69)
**Health consciousness**				
To an average degree/not so much/not at all			Ref.	Ref.
Much/very much			1.07 (0.99–1.16)	1.05 (0.97–1.14)
**Self-efficacy**				
			0.96 (0.91–1.01)	0.93** (0.88–0.98)
**Health status**				
Fair/poor/very poor				Ref.
Good/very good				1.30*** (1.20–1.41)

Results from multinomial logistic regression analyses. ^1^ = reference group *planning*; RRR = relative risk ratio; CI = confidence interval; Ref. = reference category. Significant associations shown as * = p < 0.05, ** = p < 0.01, and *** = p < 0.001

**Table 3b:** Motivational readiness for leisure-time physical activity according to health literacy and the covariates for “In activity” ^1^, n = 21,895

	Model 1	Model 2	Model 3	Model 4
	RRR (95% CI)	RRR (95% CI)	RRR (95% CI)	RRR (95% CI)
**Health literacy**				
Low	Ref.	Ref.	Ref.	Ref.
Medium	1.35** (1.11–1.63)	1.34** (1.11–1.63)	**1.21** (1.00–1.48)	**1.17** (0.96–1.42)
High	1.65*** (1.39–1.96)	1.65*** (1.39–1.96)	1.32** (1.10–1.59)	1.23* (1.02–1.48)
**Sex**				
Women		Ref.	Ref.	Ref.
Men		1.25*** (1.15–1.36)	1.41*** (1.30–1.54)	1.41*** (1.30–1.54)
**Age group** (in years)				
18–29		1.87*** (1.65–2.12)	1.93*** (1.69–2.20)	1.85*** (1.63–2.12)
30–44		Ref.	Ref.	Ref.
45–64		1.47*** (1.31–1.65)	1.37*** (1.21–1.54)	1.48*** (1.31–1.67)
65+		2.33*** (2.03–2.67)	1,87*** (1.61–2.16)	2.22*** (1.91–2.58)
**Education**				
Low		Ref.	Ref.	Ref.
Medium		**1.08** (0.94–1.23)	**1.03** (0.90–1.18)	**1.00** (0.87–1.15)
High		1.18* (1.02–1.36)	**1.04** (0.89–1.20)	**0.97** (0.83–1.13)
**Health consciousness**				
To an average degree/not so much/not at all			Ref.	Ref.
Much/very much			2.96*** (2.70–3.24)	2.86*** (2.61–3.13)
**Self-efficacy**				
			1.12** (1.04–1.19)	**1.04** (0.97–1.11)
**Health status**				
Good/very good				Ref.
Fair/poor/very poor				1.76*** (1.57–1.97)

Results from multinomial logistic regression analyses. ^1^ = reference group *planning*; RRR = relative risk ratio; CI = confidence interval; Ref. = reference category. Significant associations shown as * = p < 0.05, ** = p < 0.01, and *** = p < 0.001

### In activity (reference group: planning)

A high health literacy level was positively associated with being *in activity* (Model 1; shown in Table 3b: RRR = 1.65). This effect is weakened when controlling for health consciousness and self-efficacy (Model 3; shown in Table 3b: RRR = 1.32) and even more so when controlling for health status (Model 4; shown in Table 3b: RRR = 1.23). In the fully adjusted model, participants with a high compared to a low health literacy level are 23% more likely to be *in activity* than in *planning.*

A medium level of health literacy also showed a positive association in the crude model (Model 1: RRR = 1.35) and when controlling for socio-demographic variables (Model 2: RRR = 1.34). However, when additionally adjusting for health consciousness and self-efficacy (Model 3: RRR = 1.21) and by further adjusting for health status (Model 4: RRR = 1.17), there was no longer a significant association.

The findings showed that there is an inverse relationship between a high and medium level of health literacy (compared to a low level) and *no intention* (rather than to the *planning*). Pointing in the same direction an association was shown between a high (but not a medium) level of health literacy (compared to a low level) and being in the *in activity* group (rather than in the *planning* group). Thus, participants with a high health literacy level are more likely in a more advanced stage of motivational readiness for physical activity.

All covariates were independently associated with the phases of motivational readiness for physical activity. Being a man was associated with a higher probability of being in the *no intention* group but also of being in the *in activity* group. The highest age group (‘65+’) was shown to have a strong association with both, *no intention* and *in activity* (Tables 3a and 3b, Model 4), when compared to the age group ‘30–44’. In Model 4, a medium and high education level was associated with the probability of being in the *planning* phase compared to *no intention*, but there was no association found between the upper education levels and being in the *in activity* phase. In the fully adjusted Model 4, a strong health consciousness was associated with the *in activity* phase, whereas there was no significant relationship between health consciousness and *no intention*. According to the fully adjusted model, a higher self-efficacy was associated with a lower probability of *no intention* but no increased probability of *in activity*. A ‘good’ or ‘very good’ health status was associated with a higher probability of both, *no intention* and *in activity*.

## Discussion

Based on this large population-based sample for German adults, we observed a positive association between health literacy and motivational readiness for physical activity even if controlling for sex, age, education, health consciousness, self-efficacy, and health status. Population groups differed in their motivational readiness for physical activity depending on their level of health literacy; a medium and high level of health literacy was associated with being in the *no intention* group rather than in the *planning* group. A high level of health literacy was positively associated with being in the *in activity group* rather than in the *planning* group.

The observed positive association between health literacy and motivational readiness for physical activity is in line with the results of other studies. Aygun and Cerim [41] also observed that a higher health literacy level (assessed with the HLS-EU-Q47) was associated with being in the maintenance stage rather than in the precontemplation stage for exercising regularly, although in that study the criterion for being active was defined in a different way. The results of this study also point in the same direction as the findings of previous studies on other health behaviours, showing a positive relationship between higher health literacy and more advanced stages of change in smoking cessation behaviour [46] and between health literacy and glycaemic control in individuals with diabetes, mediated by dietary knowledge and the stages of change for healthy eating [47]. Our findings are also consistent with a recent systematic review summarizing empirical results that indicate quite consistently that higher health literacy is associated with more physical activity [30]. As the authors argue in their discussion, physical activity interventions could mitigate the negative impact of low health literacy at baseline, which could affect consciousness and motivation, as well as the ability to overcome barriers to changing physical activity habits [30].

With this in mind, it is of great interest how the relationship between health literacy and motivational readiness to change health behaviour can be explained and which dimensions of health literacy are relevant for the implementation of new habits and should be considered when designing physical activity interventions. According to Nutbeam [22], there are three different types of health literacy: functional, interactive, and critical. These types are reflected in the conceptual model underlying the health literacy instrument used in this study [20, 68]. Functional health literacy refers to the basic skills of handling health information, that is, reading competence and familiarity with relevant medical terms. It refers to the cognitive dimension of understanding e.g. in the sense of being able to follow directions of health professionals regarding medication. Interactive health literacy includes not only more advanced cognitive but social skills and allows for accessing health issues with health professionals, family, or others and applying it in different contextual conditions in order to take advice and make healthy decisions. Critical health literacy is the most advanced and involves the ability to critically evaluate health information and consciously act to create an environment that promotes one’s own health and the health of others. In this sense, the higher levels of interactive and critical health literacy include a behavioral dimension and can enable individuals to change their lifestyles and facilitate the adoption of healthy behaviours by others [22, 40]. In line with this concept, not only the cognitive (corresponding to functional health literacy), but also the behavioural dimensions of health literacy, are relevant for the motivational readiness for health behaviour change resulting in forming and implementing intentions of physical activity. In our study, only a high level of health literacy, but not a medium level, was associated with the *in activity* phase in the fully adjusted model. This suggests that a high level of health literacy corresponds to the more advanced interactive or critical type of health literacy that leads to personal empowerment to translate healthy choices into action. This observation supports Nutbeam’s suggestion that health promotion programmes (including physical activity interventions) should aim to increase the personal capacity to act autonomously on the basis of health information, and improve motivation and self-confidence to implement healthy choices [22].

It is important to reiterate that, according to the conceptual model underlying the health literacy tool used, as well as Nutbeam’s model, health literacy promotion is not only about building personal skills, but also about changing political, social and environmental conditions to facilitate the use of health information and make healthier choices. Also, while the results of this study can give a first indication that health literacy promotion is a relevant approach for health promotion in the field of physical activity, a more precise insight into dimensions of health literacy relevant for physical activity promotion can potentially be gained on the basis of physical activity-specific conceptual models of health literacy and corresponding instruments. The concepts of “physical literacy” [69, 70] and in particular of “physical activity-related health competence” should be mentioned here [71, 72]. The latter is more comprehensive and includes movement competence, control competence, and physical activity-specific self-regulation competence. It thus offers an approach to also consider the affective dimension of health literacy and provides a conceptual framework to understand more deeply the specific subdimensions of health literacy regarding physical activity.

This study focused on the association between health literacy and motivational readiness for physical activity. This association not only persists when adjusting for covariates, the results also highlighted that all covariates themselves are independently related to the stages of change. Being a man, younger aged, and with higher education appeared to be protective factors, which points in the same direction as the results of other studies [49, 59]. Interestingly, according to our study, 30–44-year-olds and women were less likely to be *in activity* and most likely to be in the *planning* phase. These results indicate an intention to change physical activity habits but points out barriers, probably due to time constraints in the context of building a family and a career in this age group often conferred to as the ‘rush hour of life’ that often particularly affects women [73]. While a medium and high education level was a protective factor against being in *no intention*, the upper education levels were not linked to the *in activity* phase. As education can be regarded as a proxy for knowledge, knowledge is one aspect of health literacy that appears to be important to move from *no intention* to *planning*, but to move to *in activity*, more than knowledge is needed in line with the comprehensive concept of health literacy, including motivation and competences [20].

Our results also showed the association between health consciousness and the probability of being *in activity* compared to *planning*; however, health consciousness was not associated with a lower probability of *no intention*. This was opposed to our expectations, as according to the transtheoretical model, the process of consciousness raising is most relevant in the earlier stages of behavioural change [74]. Possibly, health consciousness is an ongoing proactive attitude towards health, which is more relevant when motivational readiness is already advanced [75]. Also, according to Model 4 (cf. Tables 3a and 3b), a higher self-efficacy reduces the relative risk of having *no intention* of physical activity. It is not a significant predictor of being *in activity*, although according to the transtheoretical model, self-efficacy would be expected to be continuously increasing with progress through the stages of change [43]. It has been suggested to distinguish between different types of self-efficacy for the earlier (motivational) and more advanced (volitional) phases of behaviour change [76]. The present study examined general self-efficacy (confidence in their general capability of problem solving), which appeared to be significant for only the early motivational phase of behaviour change. To move to the *in activity* phase, individuals might not only need confidence in their general capability of problem solving but confidence and competence to deal with specific barriers to plan and initiate the behaviour change (volitional processes). This is in line with the above introduced concepts of physical literacy [70] or also physical activity-related health competence [71], in which motivation and confidence are considered key to engage in physical activity to translate intentions to exercise into regular physical activity throughout the course of life.

Inconsistently with other studies [49, 59], in the fully adjusted model, a ‘good’ or ‘very good’ health status was not only associated with higher motivational readiness (*in activity)* but also with the *no intention* phase. It is possible that a positive perception of one’s health status is less likely to be a trigger for a behaviour change. Additionally, when controlling for health status, the association between high health literacy and *in activity* was lower, which underlines the relevance of perceived health for the motivational readiness for physical activity.

### Practical implications

Results from our study add to the evidence that people differ in their motivational readiness for physical activity according to their health literacy level. Therefore, it seems promising to consider health literacy when designing stage tailored interventions to initiate regular physical activity, for example, by promoting the knowledge about the relationship between physical activity and physical and mental well-being. Our results support the recommendation that health promotion studies and interventions aiming to improve the level of health literacy should be undertaken simultaneously [20, 41].

Since our study shows that earlier stages of motivational readiness for physical activity are associated with a low health literacy level, while advanced stages are more likely to be linked to a high health literacy level, an approach tailored to different health literacy levels might also be helpful. A strength of stage-matched interventions is that people are met in the difficult and dynamic process of health behaviour change, which may be described as a journey [74, 77]. Interventions could be designed to include options that are accessible to people at different levels of motivational readiness for physical activity and at different levels of health literacy, for example appealing short information on the recommendations for physical activity in social media combined with instructions for everyday exercises. It may be helpful to combine existing approaches to stage-matched interventions and health literacy promotion, but these should be carefully evaluated.

Promoting health literacy involves empowering people to advocate proactively for their own health [19]. This matches with the results of this study, which, in addition to health literacy, also point to the relevance of the psychological factors of self-efficacy and health consciousness of motivational readiness to change. This suggests that both factors should be addressed in interventions to promote physical activity. Raising awareness of one’s health and supporting the feeling of being able to make healthier choices are important challenges in health promotion [78].

However, it is very important to keep in mind that health literacy is not an individual trait but depends on the fit between the provided information by society and the individuals’ ability to use it. The environmental and social setting should also be addressed in interventions to create ‘health-literate settings’ [19]. In this context, it might be worth considering what the health literacy dimension of ‘applying health information’ means in relation to physical activity when designing health-literate environments. Comprehensible and tangible health information as well as a variety of opportunities for its application, for example, through the expansion of sports fields, should be provided.

When designing health-literate settings and environments that provide possibilities to move forward in the motivational readiness for physical activity, it is important to consider different needs and barriers [79]. In this study it has been shown that some groups, for example, the 30–44-year-olds, women, or those with poorer health status, seem more vulnerable to not move from *planning* to *in activity*, which indicates the need of matched preventive offers for people with time constraints or poor health.

### Directions for further research

Our results suggest that we can increase motivational readiness for physical activity by improving health literacy. However, in order to intervene in a targeted way, we need to deepen our understanding of which dimensions of health literacy promote progress through the stages of behavioural change in physical activity. In the transition from *no intention* to *planning*, knowledge (the cognitive dimension of health literacy) may play a relevant role, for example, increasing knowledge about health benefits of physical activity in line with the process of consciousness raising that their own sedentary behaviour is problematic. For the step from *planning* to *in activity*, competence (the behavioural dimension of health literacy) to apply health information might be more important, for example, translating information on healthy training in daily decisions to exercise. Future studies should specifically investigate which and how components of health literacy and the stages of change for physical activity relate to each other.

First, further research should consider using health literacy concepts and instruments that focus specifically on physical activity itself like physical literacy [69, 70, 80] and physical activity-related health competence [71, 72]. They should enable a more detailed picture of the relationship between motivational readiness, motivation, volition, health literacy and physical activity. Second, research should use health literacy or physical literacy instruments that focus on health promotion. The short questionnaire on generic health literacy used in our study, had only some items on this domain as it also covered the domains of health care and disease prevention [53, 54]. One could expect a stronger positive association between motivational readiness for physical activity and health literacy in the domain of health promotion compared to the other domains. This might be a valuable hypothesis as a starting point for a further research question. This further research could probably reveal many specific approaches for interventions of the readiness for physical activity in leisure time. Then, intervention studies need to be carried out to investigate whether the approach of promoting health literacy within the context of stage-specific physical activity promotion measures can be effective and which methods are suitable.

In our study the chosen threshold of physical activity was quite high: only those participants engaging during leisure-time in at least 150 min at least of moderate intensity aerobic activity and did muscle-strengthening activities twice a week were classified as being active and being in the *in activity* group. In further research it could be worthy to choose a lower threshold in order to differentiate between participants that are not at all (or barely) active from persons that are active but do not meet the above-mentioned criteria. Results of this analysis could give insights on population groups that might have greater barriers to initiate physical activity and have to be addressed differently. There is also a need for further investigating on groups that have been shown to be less likely to progress into the phase of *in activity*. In order to design tailored health promotion measures, further analyses are needed that consider mediating (confounding) and moderating (interaction) factors in this relationship. It should be considered that health promotion interventions take place in real-life settings and are complex as a result. Realist approaches that investigate ‘what works for whom, under which circumstances, and why’ could be useful in this context [81, 82].

### Strength and limitations

A major strength of the study is that a large nationwide sample was studied. To our knowledge, this study is one of the first studies to explicitly examine the association between health literacy and the motivational readiness for physical activity besides Aygun et al. [41], who studied a regional sample. Despite the randomised sampling, a selection bias due to the different willingness to participate in the population is probable, which was counteracted with the help of weighting factors in the analysis. However, it was noticeable that the proportion of people with high health literacy was higher in GEDA 2014/2015-EHIS than in comparable studies [21, 29, 55]. As a result, health literacy is probably measured too positively overall, which can lead to an underestimation of the effect of health literacy on motivational readiness for physical activity. At the same time, in the present study, participants with at least one missing variable of interest were excluded, which may have led to a further selection bias. Those with missing values were on average older and more often low educated, less likely to have at least good health, and more likely to be inactive in comparison to those included in the study.

The outcome variable in the present study does not include work-related or transportation-related physical activity, although WHO states that the recommendations for physical activity can be achieved during leisure time, as well as transport or paid work or work in the household [8]. It is conceivable that, for example, people who cycle daily and have a healthy level of physical activity were classified as inactive. When interpreting the results, it should be considered that the study focused on motivational readiness for physical activity in leisure time. By excluding, for example, walking with moderate intensity in the classification of being active, the effect of health literacy on motivational readiness might be underestimated. The proportion of participants classified of being *in activity* according to this criterion is marginally different from the calculation without active transportation (23.2% instead of 21.3%).

A clear strength is that data on health literacy and motivational readiness for physical activity were both assessed with validated questions and evaluated according to an established algorithm [54, 83]. Notably, the HLS-EU-Q16 measures generic health literacy with 16 items of which only two refer indirectly on physical activity, e.g., ‘How easy or difficult do you find it to understand health warnings about behaviours such as smoking, low physical activity, or excessive drinking?’. There is an increasing interest in behaviour-specific health literacy, including physical literacy [69, 80, 84]. As described above, it could be meaningful to also explore the relationship between physical literacy and the readiness to change in order to develop stage-matched interventions [85].

Considering the motivational readiness for behavioural change, the question of how many stages should be distinguished continues to be the subject of theoretical and empirical research [86]. For the regression analyses, we combined the five stages of change into three categories, which results in a certain loss of information that was collected in the data. To answer our research question whether there is an association between health literacy and the stages of change, it was assumed to be most relevant to differentiate between those participants with no intention at all, those that were planning, and those that were in activity.

## Conclusion

Despite the limitations, we conclude that there is an association between health literacy and the stages of change for physical activity, i.e., individuals differ in their readiness to initiate change depending on their level of health literacy. Individuals with low health literacy, compared to medium and high health literacy, are at greater risk for having *no intention* to increase their level of physical activity during leisure time. When designing stage-matched interventions to promote physical activity, health literacy should therefore be addressed and strengthened. Further research is needed to deepen the understanding of which dimensions of health literacy are relevant and how they can be addressed in effective interventions of the motivational readiness for physical activity.

## Data Availability

The dataset analysed in the current study is available as public use files from the Robert Koch Institute (https://www.rki.de/EN/Content/Health_Monitoring/Public_Use_Files/public_use_file_node.html).

## List of abbreviations

WHO: World Health Organisation
GEDA 2014/2015-EHIS: German Health Update 2014/2015 and European Health Interview Survey
EHIS-PAQ: European Health Interview Survey – Physical Activity Questionnaire
HLS-EU-Q16: short form of the European Health Literacy Survey Questionnaire
ASKU: general self-efficacy short scale (Allgemeine Selbstwirksamkeit Kurzskala)
RRR: relative risk ratio
95% CI: 95% confidence interval
